# Stimulation of superoxide production increases fungicidal action of miconazole against *Candida albicans* biofilms

**DOI:** 10.1038/srep27463

**Published:** 2016-06-07

**Authors:** Kaat De Cremer, Katrijn De Brucker, Ines Staes, Annelies Peeters, Freija Van den Driessche, Tom Coenye, Bruno P. A. Cammue, Karin Thevissen

**Affiliations:** 1 Centre of Microbial and Plant Genetics, KU Leuven, Kasteelpark Arenberg 20, box 2460, 3001 Leuven, Belgium; 2 Department of Plant Systems Biology, VIB, Technologiepark 927, 9052 Gent, Belgium; 3 Laboratory of Pharmaceutical Microbiology, Ghent University, Ottergemsesteenweg 460, 9000 Gent, Belgium

## Abstract

We performed a whole-transcriptome analysis of miconazole-treated *Candida albicans* biofilms, using RNA-sequencing. Our aim was to identify molecular pathways employed by biofilm cells of this pathogen to resist action of the commonly used antifungal miconazole. As expected, genes involved in sterol biosynthesis and genes encoding drug efflux pumps were highly induced in biofilm cells upon miconazole treatment. Other processes were affected as well, including the electron transport chain (ETC), of which eight components were transcriptionally downregulated. Within a diverse set of 17 inhibitors/inducers of the transcriptionally affected pathways, the ETC inhibitors acted most synergistically with miconazole against *C. albicans* biofilm cells. Synergy was not observed for planktonically growing *C. albicans* cultures or when biofilms were treated in oxygen-deprived conditions, pointing to a biofilm-specific oxygen-dependent tolerance mechanism. In line, a correlation between miconazole’s fungicidal action against *C. albicans* biofilm cells and the levels of superoxide radicals was observed, and confirmed both genetically and pharmacologically using a triple superoxide dismutase mutant and a superoxide dismutase inhibitor N-N′-diethyldithiocarbamate, respectively. Consequently, ETC inhibitors that result in mitochondrial dysfunction and affect production of reactive oxygen species can increase miconazole’s fungicidal activity against *C. albicans* biofilm cells.

*Candida albicans*, the major fungal pathogen of humans, has the capacity to form biofilms on biotic surfaces throughout the human gastro-intestinal tract and genital area. While this fungus can cause life-threatening invasive infections in immunocompromised patients, many more individuals are confronted with superficial mucosal infections. Though the latter type of infection is often rather harmless and easy to treat, the incidence of recurrent oral and vulvovaginal candidiasis is significant. About 70% of all premenopausal woman develop vulvovaginal candidiasis at some point in their lives^1^ and repeated attacks affect at least 75 million women annually^2^. Currently the preferred treatment of such infections remains a topical azole treatment^3^.

Azoles, including imidazoles (e.g. miconazole) and triazoles (e.g. fluconazole), interfere with the biosynthesis of ergosterol by inhibiting the enzyme lanosterol 14-alpha-demethylase. Since ergosterol is a major constituent of the fungal membrane, its depletion results in growth inhibition^4^. This inhibitory effect is augmented by the accumulation of toxic ergosterol precursors^5^. Some azoles such as miconazole, induce accumulation of reactive oxygen species (ROS) in planktonic and biofilm fungal cells^6^,^7^,^8^,^9^,^10^,^11^,^12^. In contrast to other azole-type antifungals, a fungicidal effect of ROS-inducing miconazole against biofilms of several *C. albicans* strains was reported when used in high (millimolar) concentrations^10^,^11^, but the causal relationship between induction of ROS and fungicidal activity remains under debate^13^,^14^. Interestingly, targeting *C. albicans’* oxidative defence system to sustain high ROS levels reportedly enhances the fungicidal activity of ROS-inducing antifungals^6^,^11^,^13^,^15^,^16^.

Given the high tolerance of *C. albicans* biofilm cells to miconazole^14^, we aimed at obtaining more insight in the molecular pathways that are employed by the biofilm cells to resist miconazole treatment. Compounds known to affect these particular processes and pathways might result in an increased and potentially fungicidal action (at lower concentrations) of miconazole against *C. albicans* biofilm cells, which is highly desirable in the context of developing novel biofilm eradication strategies.

Gene expression profiling of drug-treated cell populations is a useful strategy to obtain new insights in the drug’s mode of action, as well as in tolerance mechanisms against the drug^17^,^18^,^19^. To our knowledge, there are currently no transcriptome datasets available of miconazole-treated *C. albicans* biofilm nor planktonically grown cells. However, a single report documents the transcriptional response of *C. albicans* biofilm cells against another azole, namely fluconazole^20^. In the latter study, the transcriptome changes in biofilm cells were studied after 30–120 min of fluconazole exposure. However, using this setup, the authors found only 5 differentially expressed genes.

In the present study, a whole-genome transcriptional analysis of miconazole-treated *C. albicans* biofilm cells was performed at 4 h and 24 h post miconazole treatment. By targeting particular miconazole-affected pathways identified via this transcriptomic approach we successfully identified compounds that increase the sensitivity of the *C. albicans* biofilm cells to miconazole. However, this potentiating effect was not observed in planktonic *C. albicans* cultures. Interestingly, the increased killing by synergistic combinations of miconazole and such specific inhibitors correlated with an increased production of superoxide radicals in the biofilm cells under aerobiosis. In line, the observed synergy between miconazole and the inhibitors under aerobic conditions was no longer apparent when biofilms were treated in anaerobic conditions, pointing to biofilm-specific miconazole tolerance pathways aimed at reducing superoxide production in the presence of oxygen. The latter was confirmed genetically using a triple superoxide dismutase mutant and pharmacologically using a superoxide dismutase inhibitor N-N′-diethyldithiocarbamate.

## Results

### Transcriptional analysis of miconazole-treated *C. albicans* biofilms

**Figure 1:**
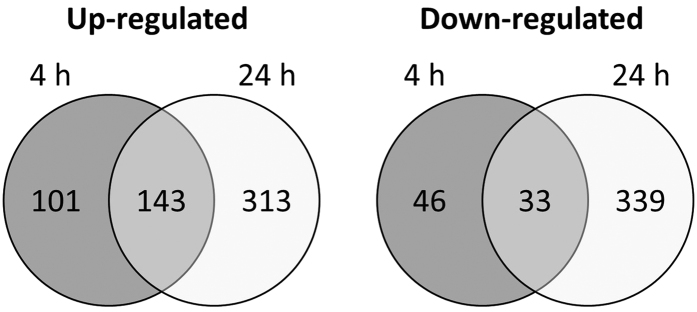
Number of significant (overlapping) differentially expressed genes 4 h and 24 h after miconazole treatment. Using EdgeR, a negative binomial distribution of the count reads was the basis to select differentially expressed genes between miconazole treated and untreated samples at both time points, with a limited false discovery rate (FDR adjusted p-value <0.01).

In order to find pathways and processes involved in generating tolerance of *C. albicans* biofilm cells against miconazole, we performed a genome-wide transcriptome analysis of *C. albicans* SC5314 biofilms treated with miconazole. First, twenty-four hour old *C. albicans* biofilms were treated for an additional 24 h with a concentration series of miconazole to determine the minimal concentration needed to reduce the metabolic activity to 50% (i.e. the minimal biofilm eradicating concentration or BEC50 = 75 μM measured with the metabolic activity dye Cell-Titre Blue (CTB); data not shown). Next, 24 h old *C. albicans* biofilms treated for 4 h and 24 h with 75 μM miconazole or mock-treatment (0.5% DMSO) were collected from three independent experiments and RNA was isolated. RNA-sequencing (RNA-seq) generated an average of 8,325,004 reads per sample of which on average 6,459,852 aligned uniquely with annotated coding sequences of the *C. albicans* SC5314 genome (Supplementary Table S1). Using EdgeR, we compared the gene expression level of each gene in the miconazole-treated samples with control samples for both time points, identifying a total of 323 and 828 differentially expressed (DE) genes at 4 h and 24 h after miconazole treatment, respectively (Supplementary Data S1). The number of overlapping up- and down-regulated genes is summarized in Fig. 1.

To examine the transcriptional response of *C. albicans* biofilms upon miconazole treatment, we first looked into the top-induced and –repressed genes (colour-indicated in Supplementary Data S1), after which we performed gene ontology (GO) analysis of the whole dataset.

Among the top-50 induced genes, expected miconazole-tolerance targets were found at both time points. More specifically, many ergosterol biosynthesis genes (*ERG6, ERG251, ERG3* and *ERG2* at both time points), genes encoding drug efflux pumps (*CDR2* and *orf19.4531* at both time points and *MDR1* only at 24 h post treatment) and a superoxide dismutase which protects against oxidative stress (*SOD5* only at 24 h post treatment) were highly induced by miconazole. These data corroborate with previous studies demonstrating a role for these genes in azole resistance, also in *C. albicans* biofilms^11^,^21^,^22^,^23^,^24^. Interestingly, genes coding for degradative enzymes such as secreted aspartic-type proteinases (*SAP3, SAP4* and *SAP6* at both time points) and lipases (*LIP5* at both time points and *LIP8* only at 24 h after treatment) were also highly induced. Induction of expression of *SAP* genes (*SAP2* and *SAP9*) was observed previously in *C. albicans* after treatment with fluconazole and this was confirmed at protein level, also for miconazole^25^. Interestingly, the expression of multiple *SAP* and *LIP* genes is upregulated in untreated *C. albicans* biofilms compared to a planktonic culture as well^26^. However, no evidence for a role of the Saps or lipases in the tolerance mechanisms against azoles or in their mode of action currently exists. Potentially, this induction is only relevant *in vivo* to invade surrounding tissue and escape the drug treatment. Also the expression of many genes encoding cell wall proteins (*PGA31*, *PGA37*, *orf19.675, PGA23* and *SIM1* at both time points*, RHD3* and *SCW11* only at 4 h post treatment and *PGA22* only at 24 h post treatment) and of a putative nicotinic acid transporter (*TNA1* at both time points) was highly induced, while these genes have not been linked to miconazole’s mode of action or tolerance pathways in biofilm cells. The biofilm transcriptional response to miconazole treatment suggested a broad disturbance of energy metabolism, illustrated by the high induction of genes encoding the key glyoxylate enzyme isocitrate lyase (*ICL* at both time points) and several fermentation enzymes *ADH3* (only at 4 h post treatment) and *ADH4* and *ADH5* (only at 24 h post treatment). Fermentation was recently reported to be specifically induced during *C. albicans* biofilm formation compared to stationary phase planktonic cells, possibly because of the hypoxic environment within *C. albicans* biofilms^27^,^28^, which might be stimulated when treated with ROS-inducing compounds such as miconazole. Moreover, multiple amino acid metabolism genes (*LEU1* and *MET3* at both time points, *GDH3* only at 4 h post treatment and *CAR2* only at 24 h post treatment) were highly induced. In addition, a gene encoding a putative secreted acid sphingomyelin phosphodiesterase (*orf19.4707*, induced at both time points), *IPT1 (*induced at 4 h post treatment only), which catalyses the synthesis of the most abundant sphingolipid mannose-(inositol-P)2-ceramide and *LCB4* (induced at 24 h post treatment only), encoding a sphingosine kinase, were induced (Supplementary Data S1). Interestingly, both *C. albicans* and *Saccharomyces cerevisiae* mutants affected in sphingolipid biosynthesis, including *ipt1* and *lcb4*, portray altered miconazole-susceptibility, confirming a possible role for these sphingolipids in miconazole’s mode of action^29^,^30^.

Whereas a large overlap was observed between the top genes induced at 4 h and 24 h after miconazole treatment, we found more differences between both time points when comparing the top-repressed genes (Supplementary Data S1). The most notable down-regulated genes were those involved in arginine biosynthesis (*CPA1, CPA2, ARG1, ARG3, ARG4* and *ARG8* and to lesser extent also *ARG11* and *ARG5,6*) which was only observed at 24 h post miconazole treatment. Also two putative gamma-aminobutyric acid transaminase encoding genes (*UGA1* and *UGA11*) are only repressed at 24 h post miconazole treatment. For both processes, no obvious link with miconazole actions has been described. At 4 h post miconazole treatment, the repression of genes involved in iron homeostasis (*FRE7* and *FRE30*) was evident, and this downregulation was still ongoing at 24 h post treatment (although at lower reduction levels). Interestingly, the catalase-encoding gene *CAT1*, conferring resistance to oxidative stress, was also repressed at both time points (most pronounced at 4 h post treatment), which is in line with our previous hypothesis linking the miconazole-induced ROS production to miconazole’s direct inhibition of ROS-degrading enzymes such as catalase^7^. Other top-repressed genes, which are significantly differentially expressed at both time points, include *ARO10* (aromatic decarboxylase), *FDH1* (formate dehydrogenase), *GLT1* (a putative glutamate synthase), *MSH6* (potentially involved in mismatch repair via interacting selectively and non-covalently with double-stranded DNA containing a single base insertion or deletion), *NGT1* (N-acetylglucosamine-specific transporter) and the transcriptional regulator of cell cycle gene expression, *NRM1* (Supplementary Data S1).

### Gene ontology analysis

**Figure 2:**
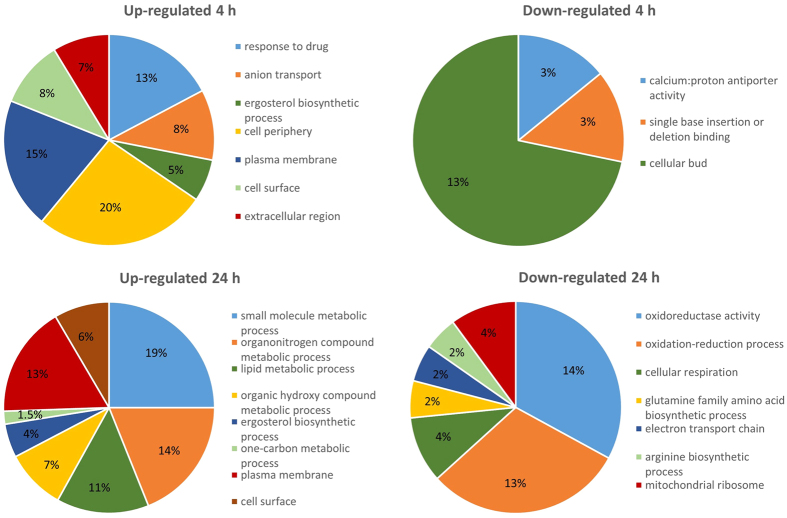
Distribution of major significantly overrepresented GO categories within the significantly up- or down-regulated gene lists at 4 h and 24 h post miconazole treatment, respectively. The percentages represent the relative amount of differentially expressed genes annotated to the respective GO category within the list of selected differentially expressed genes. Note that the sum of all represented GO categories in each pie chart is not equal to 100% since some genes belong to multiple GO categories and many of the differentially expressed genes are unannotated or annotated to GO categories that were not significantly overrepresented. Significant overrepresented GO categories were determined using the GO Term Finder at the Candida Genome Database (CGD) website (FDR adjusted p-value < 0.05).

In parallel to a gene-specific expression analysis of the top-induced and -repressed genes, we also focused on the entire gene expression profile by performing a gene ontology (GO) enrichment analysis. This enables us to identify groups of differentially expressed genes representing different biological processes, molecular functions or cellular components (Supplementary Data S2). In Fig. 2 we depicted the most unique GO categories overrepresented in the corresponding gene lists.

At 4 h post miconazole treatment, genes coding for sterol biosynthesis and related GO categories were significantly overrepresented among the induced genes, as well as those coding for ion transport and genes encoding proteins located in the extracellular region, cell surface and plasma membrane (Fig. 2 and Supplementary Data S2). Gene groups significantly over-represented in the down-regulated gene lists at 4 h post miconazole treatment included those correlated with calcium:proton antiporter activity, single base insertion or deletion binding and cellular bud (Fig. 2 and Supplementary Data S2).

At 24 h post miconazole treatment, sterol biosynthesis and related GO categories were again among the most pronounced induced GO categories. The GO category ‘other small molecule biosynthetic processes’ was also significantly induced and the concomitant induction of the organo-nitrogen compound metabolic process suggests these small molecules are mainly related to metabolism of amino acids, although pyridoxine biosynthesis genes were also represented in this category (*PDX3*, *SNO1* and *RKI1*). Additionally, one-carbon metabolic process (mainly genes involved in tetrahydrofolate interconversion) was significantly induced at 24 h post miconazole treatment. Regarding cellular components, many genes encoding for proteins located on the cell surface and plasma membrane were induced. Gene groups significantly over-represented in the down-regulated gene lists at 24 h post miconazole treatment included the glutamine family amino acid biosynthetic process (linked to synthesis of glutamate, glutamine, arginine and proline of which mainly arginine biosynthetic genes were down-regulated at 24 h post miconazole treatment), oxidation-reduction process and cellular respiration (including eight components of the respiratory electron transport chain (ETC), namely *COX6, COX9, CYT1, RIP1, QCR2, QCR7, QCR9* and *orf19.913.2*). Also the GO categories related to molecular function and cellular component illustrate the repression of the ETC (Fig. 2 and Supplementary Data S2). Finally, the GO analysis revealed down-regulation of genes encoding multiple mitochondrial ribosomal constituents (*MRP17, MRP20, MRPS9, MRPL10, MPRL36, MRPL37, orf19.4751, orf19.1662, orf19.3348, orf19.3022, orf19.585, orf19.2639, orf19.688: orf19.549 and orf19.6136*). This repression could additionally lead to reduced respiration (including reduced ETC activity), the core function of proteins synthesized by mitochondrial ribosomes.

The apparent transcriptional downregulation of components of the ETC by miconazole in *C. albicans* biofilm cells hinted us to compare the current RNA-seq dataset (24 h post miconazole treatment) with a transcriptome study of *C. albicans* cells growing in hypoxic conditions^31^. Forty-two out of 245 hypoxia-induced genes (17%) were also induced in miconazole-treated *C. albicans* biofilms. Among these genes were the transcriptional regulator *UPC2*, many ergosterol biosynthesis genes (*ERG1, ERG3, ERG5, ERG11* and *ERG251*), the alcohol dehydrogenase encoding gene *ADH5* and the transcriptional regulator gene *RAS2*. On the other hand, 22 out of 164 hypoxia-repressed genes (13%) were also significantly down-regulated in miconazole-treated *C. albicans* biofilms. Many of the common down-regulated genes were involved in oxidative respiration (*TYE7, QCR2, CYT1, QCR7, RIP1, orf19.4016, NDH51*), arginine biosynthesis (*CPA1, CPA2, ARG11* and *ARG5,6*) and Fe homeostasis (*FRE7, FTR2* and *orf19.7077*). Interestingly, the similarity between the hypoxic condition and azole treatment was previously described for planktonic *C. albicans* cultures upon ketoconazole addition (which is also an imidazole, like miconazole)^31^.

Validation of the RNA-seq data was performed for both time points by qRT-PCR on a selection of 16 genes representing different biological processes that were transcriptionally affected, as described above (Supplementary Fig. S1). The resulting logFC values obtained by qRT-PCR were completely in line with the normalized logFC values from the RNA-seq data calculated with EdgeR (Pearson correlation coefficient = 0.97), thereby confirming the observed gene expression patterns in the RNA-seq data as basis for subsequent analysis (Supplementary Fig. S1).

### Miconazole combination testing reveals biofilm-specific synergies

Processes and pathways affected by miconazole in *C. albicans* biofilms, as determined by the transcriptome analysis, can lead to the identification of compounds that affect the sensitivity to miconazole. Inhibitors or inducers of such pathways may act synergistically or antagonistically in combination with miconazole against *C. albicans* biofilms, depending on the involvement of the pathway in miconazole’s mode of action or biofilm tolerance against this compound. To this end, 17 known inhibitors and inducers of miconazole-affected pathways or compounds that complement the repression of a certain pathway were selected. Their potential synergistic or antagonistic activity with miconazole against 24 h old *C. albicans* biofilms was assessed via checkerboard analysis and calculation of the fractional inhibitory concentration index (FICI) based on metabolic activity determination using CTB. Table 1 categorizes their action as synergistic or indifferent in combination with miconazole against *C. albicans* biofilms. Because of the large amount of identified differentially expressed genes and pathways, we were unable to tackle all of them by an appropriate inhibitor or inducer. Consequently, we focused on well-known and easily available compounds (Table 1).

**Figure 3:**
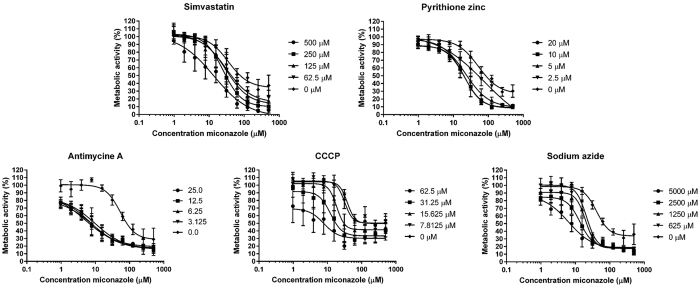
Metabolic activity of *C. albicans* biofilms treated with a combination of miconazole and simvastatin, pyrithione zinc, CCCP, antimycin A and sodium azide. The control curve of miconazole alone is represented by diamonds, whereas combinations of miconazole with decreasing concentrations of other compound are indicated by circles, squares, triangles and reverted triangles. Data are mean ± SEM of five (n = 5) biologically independent experiments (each consisting of three repeats), determined with the metabolic activity dye CTB.

Five inhibitors resulting in synergistic activity with miconazole (simvastatin, pyrithione zinc, antimycin A, CCCP and sodium azide) were selected for further analysis. A more detailed representation of the remaining metabolic activity in *C. albicans* biofilms after checkerboard analysis of the combined treatment of these inhibitors with miconazole is shown in Fig. 3. All five selected inhibitors were able to reduce the minimum miconazole concentration necessary to result in 50% residual metabolic activity of biofilm cells (BEC50) at least 4.4-fold (Table 2). The most pronounced synergistic effect with miconazole was obtained with antimycin A (FICI = 0.10). Applied at a concentration of 3.125 μM, antimycin A was able to reduce the BEC50 of miconazole almost 11-fold (Table 2). Note that control curves of miconazole (Fig. 3) and corresponding BEC50 values (Table 2) may vary among the different experiments. Therefore, FICI calculations for the five selected inhibitors were based on BEC50 values as determined within the experiments involving the compound that is being analysed.

**Figure 4:**
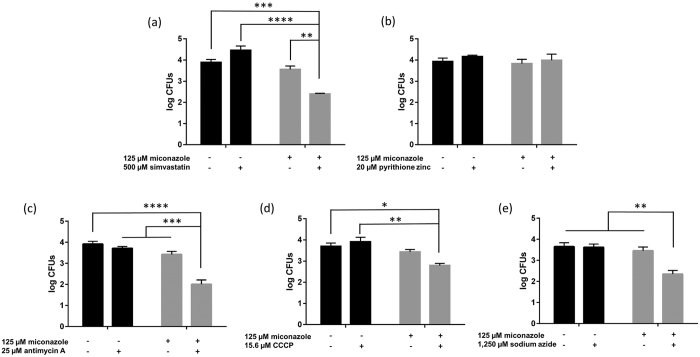
Number of cfus per *C. albicans* biofilm in the presence and absence of miconazole and/or **(a)** simvastatin, **(b)** pyrithione zinc, **(c)** antimycin A, **(d)** CCCP and **(e)** sodium azide. Black bars represent untreated biofilms or biofilms treated with 500 μM simvastatin, 20 μM pyrithione zinc, 25 μM antimycin A, 15.6 μM CCCP and 1,250 μM sodium azide alone while grey bars represent biofilms additionally treated with 125 μM miconazole. Data presented are the mean and SEM of at least three (n ≥ 3) independent experiments (each consisting of three replicates), measured by cfu counting. Two-way ANOVA statistical analysis with Tukey’s correction for multiple comparisons was performed to assign significant differences between treatments (* = p < 0.05, **p < 0.01, ***p < 0.001, ****p < 0.0001).

Of importance, one should carefully evaluate the data that measures biofilm metabolic activity (such as the CTB metabolic activity dye used in this study) in the presence of inhibitors of the ETC. Treatment of biofilms with ETC inhibitors will result in reduction of signal in such assays and control treatments should always be taken into account (miconazole + ETC inhibitor versus ETC inhibitor alone) as done in our FICI calculation. Moreover, we also investigated whether the five selected inhibitors are effective in increasing the fungicidal activity of miconazole against *C. albicans* biofilm cells by counting colony forming units (cfu) (Fig. 4). The applied inhibitor concentrations were selected to have no significant antibiofilm activity on their own, based on the checkerboard analysis (Fig. 3).

Untreated biofilms contained almost 10^4 ^ cfus/biofilm and the miconazole-only treatment (125 μM) did not significantly affect this number (<0.5 log units). Significant reductions in cfus compared to the control treatment (no miconazole and no additional inhibitor) could only be observed for the combined action of 125 μM miconazole with 500 μM simvastatin (97%), 25 μM antimycin A (99%), 15.6 μM CCCP (87%) or 1.25 mM sodium azide (95%). The largest potentiating effect was observed for antimycin A, which was able to increase the miconazole fungicidal capacity by 26-fold (corresponding with a reduction of almost 2 log units compared to no treatment). The combination of 125 μM miconazole and 20 μM pyrithione zinc did not result in additional reduction of cfus in comparison with single miconazole treatment (Fig. 4). Hence, only the synergistic activity of miconazole and simvastatin or ETC inhibitors on the metabolic activity of *C. albicans* biofilm cells translate to induced killing of these cells.

To investigate whether the synergistic activity of the five selected inhibitors with miconazole is biofilm-specific, we also assessed the activity of the above-described synergistic combinations in checkerboard assays on planktonically growing *C. albicans* cultures (Supplementary Fig. S2). While no additional activity of miconazole in combination with four of the inhibitors (simvastatin, pyrithione zinc, antimycin A and sodium azide) in these planktonic conditions could be observed, an antagonistic effect of the ETC inhibitor CCCP on miconazole’s action could be seen, as confirmed by FICI calculation (FICI = 6.63 for 12.5 μM CCCP), which is in contrast with the effect on *C. albicans* biofilms. Indeed, 12.5 μM CCCP was able to increase the MIC50 (i.e. the minimal inhibitory concentration reducing planktonic cell growth with 50%) of miconazole by nearly 6-fold (Supplementary Fig. S2). It appears that the selected inhibitors act synergistically with miconazole against biofilm-specific tolerance mechanisms.

### Boosting up miconazole’s ROS production in *C. albicans* biofilms by synergistic compounds results in increased fungicidal activity

**Figure 5:**
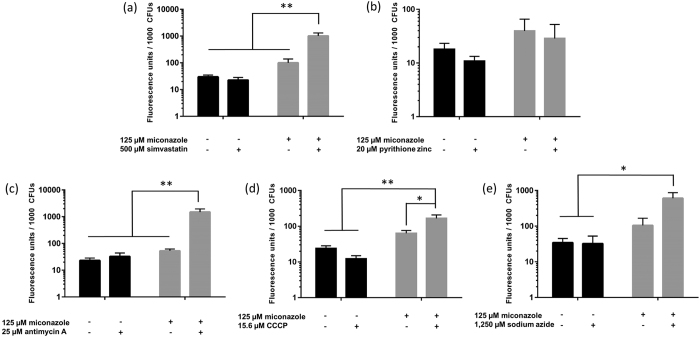
Accumulation of superoxide radicals in *Candida albicans* biofilms in the presence and absence of miconazole and/or **(a)** simvastatin, **(b)** pyrithione zinc, **(c)** antimycin A, **(d)** CCCP and € sodium azide. Superoxide production is expressed as fluorescence per 1000 cfus assessed by cfu counting. Black bars represent untreated biofilms or biofilms treated with 500 μM simvastatin, 20 μM pyrithione zinc, 25 μM antimycin A, 15.6 μM CCCP and 1250 μM sodium azide only while grey bars represent biofilms additionally treated with 125 μM miconazole. Data presented are the mean and SEM of at least three (n ≥ 3) independent experiments (each consisting of three replicates). Two-way ANOVA statistical analysis with Tukey’s correction for multiple comparisons was performed to assign significant differences between all treatments (*p < 0.05, **p < 0.01).

The fungicidal activity of antifungal compounds has often been associated with the production of ROS^14^. Therefore, we assessed whether the observed synergistic effect between miconazole and the five selected inhibitors led to increased production of superoxide radicals, using dihydroethidium staining. As the conversion of this dye depends on the number of live cells in the biofilm, results were normalized to the number of cfu/biofilm (Fig. 5).

Treatment of *C. albicans* biofilms with miconazole (125 μM) resulted on average in a 3-fold increase of the basal superoxide accumulation of untreated biofilm cells (Fig. 5). Interestingly, our data show that all combinations with fungicidal capacity against *C. albicans* biofilms (miconazole in combination with simvastatin or ETC inhibitors) cause additional superoxide production compared to treatment with the single compounds. The highest superoxide induction was observed for miconazole in combination with 25 μM antimycin A (28-fold increase compared to treatment with miconazole alone) (Fig. 5), which corresponds with the largest reduction in cfus caused by miconazole in combination with this compound (Fig. 4). On the other hand, combined addition of pyrithione zinc (20 μM) and miconazole does not induce an increase in superoxide levels in the *C. albicans* biofilms compared to miconazole treatment alone (Fig. 5), which again is consistent with the lack of effective fungicidal capacity of this combination (Fig. 4). Using the above data, we find a tight correlation between the fold reduction in cfu versus the fold increase in superoxide levels for biofilms treated with miconazole only or a combination treatment, which is confirmed by the Pearson correlation coefficient (0.95; *p* value = 0.0144; Supplementary Fig. S4).

To validate these findings further, we assessed the antibiofilm capacity of miconazole, antimycin A and the combination of both compounds in anaerobic conditions. Note that biofilms were grown in standard aerobic conditions for these tests as done for all previous experiments and that only the treatment was performed in absence of oxygen. In these conditions, oxygen cannot serve as electron acceptor for the ETC and no mitochondrial ROS are produced. Therefore, we hypothesized that addition of antimycin A has no further effect on miconazole activity. Indeed, no significant combinatory fungicidal effect of antimycin A and miconazole, compared to miconazole treatment alone, is present in anaerobic conditions (Supplementary Fig. S3). Surprisingly, the effect of miconazole alone was more pronounced in anaerobic conditions (significant reduction in cfus of 0.8 log unit) compared to aerobic treatment. This observation was confirmed by metabolic staining of *C. albicans* biofilms treated with a concentration series of miconazole in both aerobic and anaerobic conditions (Supplementary Fig. S3).

### Superoxide production is crucial for miconazole’s fungicidal action against *C. albicans* biofilms

**Figure 6:**
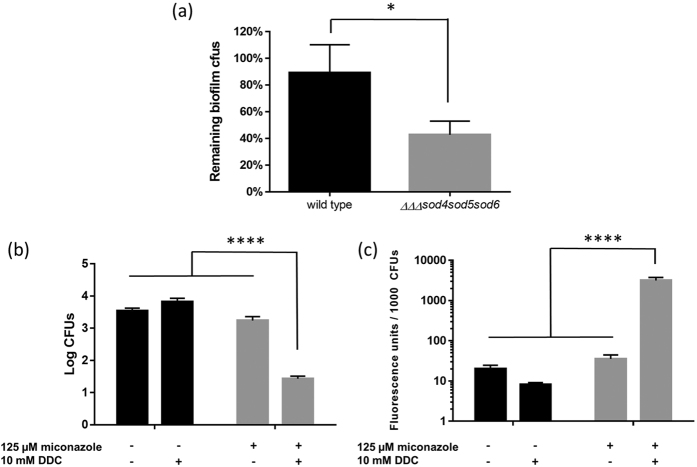
Involvement of superoxide dismutases in *C. albicans* biofilm tolerance to miconazole. **(a)** Percentage remaining biofilm cfus in wild type (CA-IF100) and triple *sod* mutant (ΔΔΔ*sod4sod5sod6)* after treatment with 30 μM miconazole. A two-tailed paired Students *t*-test was performed to assign significant differences between the two strains (*p < 0.05). **(b)** Number of cfus per *C. albicans* SC5314 biofilm and **(c)** concomitant accumulation of superoxide radicals in the presence and absence of miconazole and/or DDC. Black bars represent untreated biofilms or biofilms treated with 10 mM DDC alone while grey bars represent biofilms additionally treated with 125 μM miconazole. Data presented are the mean and SEM of four (n = 4) independent experiments (each consisting of three replicates). Two-way ANOVA statistical analysis with Tukey’s correction for multiple comparisons was performed to assign significant differences between treatments (****p < 0.0001).

To confirm the link between superoxide induction and miconazole’s fungicidal capacity we explored the role of superoxide dismutases (Sods), enzymes that rapidly convert toxic superoxide into molecular oxygen in healthy *C. albicans* cells^32^,^33^. Six different Sods are present in *C. albicans* among which four Cu,Zn-containing superoxide dismutases (Sod1, Sod4, Sod5, and Sod6)^32^ that can be inhibited using the Cu,Zn-Sod inhibitor N,N′-diethyldithiocarbamate (DDC), which chelates copper^34^. Our transcriptomic data revealed increased expression of *SOD5* and *SOD6* (Supplementary Table S2) at 24 h after miconazole treatment, suggesting their involvement in protection against these oxygen radicals. Indeed, a triple mutant in which both genes (and *SOD4*) were deleted, was hypersusceptible to miconazole compared to the wild type strain (Fig. 6a). Interestingly, no difference was observed in miconazole susceptibility between planktonically growing wild type and ΔΔΔ*sod4sod5sod6 C. albicans* cells as evidenced by the respective MIC values (MIC50 = 0.1 μM for both the triple *sod* mutant and wild type *C. albicans*; data not shown), confirming the biofilm-specific action of miconazole-induced superoxide. Finally, addition of the Sod inhibitor DDC (thereby mimicking the protein profile of the triple *sod* mutant) lead to increased fungicidal activity of miconazole against *C. albicans* biofilms, which correlated with the rise in superoxide production (Fig. 6b,c).

## Discussion

Miconazole is one of the most commonly recommended and effective topical antifungal treatments of e.g. vulvovaginal candidiasis^3^. In 80–90% of patients who complete the therapy, treatment with such azoles results in relief of symptoms and full elimination of fungal burden. Unfortunately, approximately 10–20% of women will be victim of complicated vulvovaginal candidiasis, requiring special therapeutic needs^3^. Also oral thrush, manifested by *C. albicans*, is often treated with miconazole, but recurrence is frequent, leading to consideration of other treatments^35^. Most pathogenesis studies suggest recurrence to be linked to host immune defects, however, a role for biofilm formation during both vaginal and oral *C. albicans* infections and the recurrence thereof is suggested^36^,^37^.

In this study, we analysed the transcriptome of *C. albicans* biofilms treated with miconazole aiming at gaining further insight in miconazole’s activity against biofilm cells and in biofilm tolerance mechanisms against this drug. Via RNA-seq, we identified a variety of differentially expressed genes and pathways, some of which confirmed previously observed miconazole actions (e.g. induction of *ERG* genes and genes encoding drug efflux pumps)^21^,^22^,^23^,^24^, while others are unreported.

Members of the *ERG* gene family code for ergosterol biosynthesis enzymes and their induction probably results in reduced efficacy of miconazole, which targets the lanosterol 14-alpha-demethylase encoded by *ERG11*. Induction of several *ERG* genes has been reported during biofilm development^38^, but contradictory, it was also reported that biofilm cells contain less ergosterol in their cell membranes compared to free-living cells, which possibly reduces the efficiency of azole antifungals affecting biosynthesis of this compound^22^. Genes encoding efflux pumps have also been reported to be upregulated during biofilm formation and development, which might explain the early acquisition of drug resistance^23^. However, *C. albicans* mutant strains deficient in efflux pumps were still fluconazole-resistant during biofilm growth, indicating that the presence of efflux pumps is not sufficient to account for the observed fluconazole resistance in *C. albicans* biofilms^22^,^23^. Interestingly, one study reports on a relationship between upregulation of the efflux pump gene *MDR1*, fluconazole resistance and uncoupled oxidative phosphorylation in *C. albicans*^39^, while fluconazole hypersusceptibility has also been observed in *C. albicans* mitochondrial ETC complex I mutants^40^. The latter was confirmed by the use of ETC inhibitors (including antimycin A), resulting in increased sensitivity to fluconazole. The authors suggest that the hampered ATP production results in down-regulation of efflux genes and concomitant azole susceptibility^40^. The down-regulation of calcium:proton antiporter activity (represented by *VCX1* and *orf19.7670*) is probably related to the role of the calcineurin pathway in resistance against azoles^41^, which is reported to inhibit Vcx1-dependent H^+^/Ca^2+^ exchange in *S. cerevisiae*^42^.

Many of the genes repressed at 24 h after miconazole treatment are involved in cellular respiration, which has not been previously observed in miconazole-treated *C. albicans*. It was previously reported that miconazole-induced ROS was dependent on a functional citric acid cycle and respiratory ETC in *S. cerevisiae* and increased respiration was confirmed in planktonically growing *C. albicans* when treated with miconazole^12^, which is in contrast to our data on miconazole-treated *C. albicans* biofilms. According to the GO analysis, other important significantly affected biological processes unrelated to known miconazole targets are those involving organo-nitrogen compound metabolism and one carbon metabolic processes (induced at 24 h after treatment), single base insertion or deletion binding (repressed at 4 h after treatment) and arginine amino acid biosynthesis (repressed 24 h after treatment). When grown under certain conditions (e.g. miconazole stress), normal nutrition might be impeded and therefore other carbon sources become essential for growth and metabolism of the fungus. These can include amino acids, fatty acids and carboxylic acids^43^. Together with the observed repression of the ETC, this metabolism shift explains for a large part the high amount of differentially expressed genes and overlap with hypoxia-induced gene expression changes^31^.

We selected 17 inhibitors or inducers of interesting pathways based on the GO analysis and the top-affected genes and assessed whether these compounds acted synergistically or antagonistically in combination with miconazole against *C. albicans* biofilms. The action of five inhibitors (affecting three different targets) could be defined as synergistic (FICI < 0.5) in combination with miconazole against *C. albicans* biofilms. The pathways or features affected by these inhibitors were ergosterol biosynthesis, Fe/S bonds and the ETC. The lack of combination effect with miconazole for several of the selected compounds was not totally unexpected. On one hand, it is possible that the identified pathway is not involved in miconazole’s mode of action or biofilm tolerance against the compound. On the other hand, the biofilm structure might prevent the compound from reaching its target in the *C. albicans* cells.

The first compound, the HMG-CoA reductase inhibitor simvastatin, is a cholesterol-lowering drug for which antifungal activity has been described previously^44^. Both statins and azoles target the ergosterol biosynthesis pathway in *C. albicans*. Statins act more upstream compared to azoles, potentially depleting the fungal cells from alternative sterols and sterol precursors that could be inserted in the fungal membrane upon azole treatment^45^. Interestingly, synergy between simvastatin and azoles has been described for planktonically growing yeast cultures^46^ as is the case for other statin-azole combinations^47^. However, from our data a synergistic activity between miconazole and simvastatin was biofilm-specific as this could not be confirmed in planktonic conditions. The potential of statins in systemic antifungal therapy is debated, due to the low concentrations that can be reached in blood. Moreover, the combination of statins with azole antifungals is questionable since azoles are potent inhibitors of CYP enzymes, leading to arrested metabolism of many statins in the liver^44^.

The second compound, pyrithione zinc, is used in the treatment of seborrheic dermatitis^48^. Transcriptome analysis of the effect of sub-lethal concentrations of pyrithione zinc on *Saccharomyces cerevisiae* identified the strong induction of genes related to iron transport, and many of the strongly downregulated genes are related to the biosynthesis of cytochrome (heme). More recently, it was reported that pyrithione zinc acts through an increase in cellular copper levels that leads to loss of activity of Fe/S cluster-containing proteins^49^. Such Fe/S clusters are present in many ETC proteins (repressed at 24 h after miconazole treatment) and in 3-isopropylmalate dehydratase (encoded by *LEU1*, which is one of the top-induced genes at both 4 h and 24 h after miconazole treatment). The observed synergistic effect between pyrithione zinc and miconazole (which was only effective in inhibition of metabolic activity, not in effectively killing the *C. albicans* biofilms) could be the consequence of zinc pyrithione’s action on these types of enzymes. Interestingly, several synergistic combinations of pyrithione zinc with azoles have been described and some of these are on the market for the treatment of fungal topical infections. For example, combinations between ketoconazole or climbazole and pyrithione zinc have proven effective to treat seborrheic dermatitis, a fungal skin infection condition^50^.

A similar mechanism might be the basis for the synergistic fungicidal effect of miconazole in combination with the last three compounds (antimycin A, CCCP and sodium azide), known inhibitors of the ETC. Antimycin A binds to the quinone reduction site of complex III, thereby inhibiting the oxidation of ubiquinol in the ETC^51^. The inhibition of this reaction disrupts the formation of the proton gradient across the inner membrane. Moreover, toxic ROS are formed^52^. Sodium azide blocks the mitochondrial respiratory chain at complex IV and this action reportedly decreases cellular superoxide levels^53^, although augmentation of ROS levels has been reported as well after treatment of different cell types with inhibitors of complex IV^54^. In contrast, the protonophore CCCP does not act on one particular ETC complex, but it generally enables protons to cross lipid bilayers, thereby separating the flow of electrons and the pumping of H^+^ ions. The latter implies that the energy from electron transfer cannot be used for ATP synthesis^55^. Also for CCCP treatment both induction and reduction of ROS levels has been reported^56^,^57^.

We further attempted to unravel the role for ROS in the fungicidal combinations of miconazole and ETC inhibitors antimycin A, CCCP and sodium azide, by measuring the amount of superoxide radicals present after treatment. In addition, we included simvastatin and pyrithione zinc in this assay. Only the combinations that were able to effectively kill *C. albicans* biofilms (miconazole in combination with simvastatin, antimycin A, CCCP or sodium azide, as validated by cfu counting) showed a significant increase in superoxide production compared to single compound treatment. In contrast, the synergistic combinatory effect of pyrithione zinc with miconazole, which was only observed after metabolic staining and not confirmed by cfu counting, was not associated with increased superoxide levels. Moreover, a significant correlation was observed between the fold reduction in cfus versus fold increase in superoxide levels for *C. albicans* biofilms treated with miconazole only or treated with miconazole and inhibitor.

It was previously described that miconazole induces an oxidative-damage pathway in planktonically growing *C. albicans* cultures leading to cell death as a result of the production of ROS via the citric acid cycle and respiratory chain^12^. Our data of miconazole-treated *C. albicans* biofilms points to an opposite effect occurring in sessile structures. Miconazole treatment results in down-regulation of the citric acid cycle and the respiratory chain and it appears that miconazole’s antibiofilm effect is enhanced by perturbing the latter. Previous biofilm transcriptomic and metabolomic profiling studies have demonstrated that mitochondrial respiration is down-regulated in mature *C. albicans* biofilms compared to planktonic growth^28^,^58^,^59^, suggesting this might be part of a protective mechanism for *C. albicans* biofilms as we show for miconazole in this study. The observed down-regulation of cellular respiration in the miconazole-treated *C. albicans* biofilms in this study suggests that these cells are attempting to halt toxic ROS production when treated with miconazole, a feature possibly unique to biofilm cells (in contrast with planktonic cultures), making them more tolerant to miconazole action. A similar observation was previously made in tobramycin-treated biofilms of the Gram-negative bacterium *Burkholderia cenocepacia*^60^. Compounds that restore the ROS-inducing capacity of miconazole appear to act synergistically with miconazole against *C. albicans* biofilms and reduce the living population within the biofilm. The ETC inhibitors probably succeed in this action by causing an imbalance in electron transport resulting in increased ROS production (and therefore increased fungicidal activity) by miconazole. Additionally, also inhibition of sterol precursor biosynthesis (by simvastatin) in combination with miconazole appears to synergistically increase superoxide induction and fungicidal capacity. In line, when tested under anaerobic conditions, the combination of antimycin A and miconazole had no synergistic activity against *C. albicans* biofilms. The crucial role of superoxide in the antibiofilm activity of miconazole was illustrated by the increased miconazole-sensitivity of the ΔΔΔ*sod4sod5sod6* mutant, which is hampered in detoxification of superoxide. Moreover, combination treatment of *C. albicans* biofilms with miconazole and the Sod inhibitor DDC, confirmed the mutant phenotype. Note that DDC was previously reported to also reduce the levels of persister cells within miconazole-treated *C. albicans* biofilms^11^.

In conclusion, our results show that the above described synergistic combinations (under aerobic conditions) between miconazole and simvastatin, pyrithione zinc or ETC inhibitors are biofilm-specific and lead in case of simvastatin and ETC inhibitors to increased killing of *C. albicans* biofilm cells. Hence, inhibition of biofilm-specific miconazole-affected pathways seems a promising route for the rational design of fungicidal miconazole combinations against *C. albicans* biofilms.

## Materials and Methods

### Strains and chemicals

*C. albicans* strains SC5314^61^, the homozygous triple deletion mutant in *SOD4*, *SOD5* and *SOD6* (ΔΔΔ*sod4sod5sod6* mutant) and the corresponding isogenic wild-type strain, CA-IF100^33^, were grown routinely on YPD (1% yeast extract, 2% peptone (International Medical Products, Belgium) and 2% glucose (Sigma-Aldrich, USA)) agar plates at 30 °C. RPMI 1640 medium (pH 7.0) with L-glutamine and without sodium bicarbonate was purchased from Sigma-Aldrich and buffered with MOPS (Sigma-Aldrich). Stock solutions of miconazole (Sigma-Aldrich) were prepared in DMSO (VWR International, Belgium). Other chemical compounds were purchased at Sigma-Aldrich (pyrithione zinc, antimycin A, CCCP, sodium azide, chlorosulfuron, myriocin, saquinvair mesylate, orlistat, itaconate, disulfiram, N-acetyl-D-glucosamine, sodium dichloroacetate and DDC) or TCI Europe (simvastatin and trimethoprim). Nicotinic acid, L-arginine and L-leucine were purchased at VWR International.

### Transcriptomic experiment

An overnight culture of *C. albicans*, grown in YPD, was diluted to an optical density of 0.1 (approximately 10^6 ^cells/mL) in RPMI 1640 medium and 500 μL thereof was added to the wells of a 12-well polystyrene microtiter plate (Greiner Bio-One). After 1 h of adhesion at 37 °C, the medium was aspirated and biofilms were washed with 500 μL phosphate buffered saline (PBS) to remove non-adherent cells, followed by addition of 500 μL RPMI 1640 medium. Biofilms were allowed to grow for 24 h at 37 °C. Miconazole (75 μM in RPMI 1640 medium) was then delivered to the biofilms (0.5% DMSO background). Biofilm samples were collected at 4 and 24 h after miconazole treatment for RNA isolation. Measurement of metabolic activity was performed on biofilms grown in the same conditions after washing wit PBS. To this end, 500 μL Cell-Titre Blue (CTB; Promega, USA)^62^ diluted 1/10 in PBS was added to each well. After 1 h of incubation in the dark at 37 °C, fluorescence was measured and percentage of metabolically active biofilm cells was calculated as described before^63^.

### RNA isolation

Before RNA isolation, biofilm material was washed with ice cold PBS, scraped of the bottom of the plate and washed 3 times more with ice cold PBS. RNA was isolated using a hot-phenol protocol as described previously^64^, combined with mechanical disruption using acid-washed glass beads (Sigma-Aldrich) in the Precellys 24 (Bertin technologies; 2 × 45 s with 15 s break at 6,000 rpm). Control of the quality and integrity of the RNA was done with the NanoDrop ND-1000 spectrophotometer (NanoDrop Technologies, USA) and the BioAnalyzer (Agilent, USA).

### Library preparation, RNA-sequencing and data analysis

Library preparation for RNA sequencing was performed using the Illumina (USA) TruSeq kit and sequencing was performed in one lane of an Illumina HiSeq2000 sequencer using the HiSeq Reagent Kit (Illumina, USA) according to the manufacturer’s instructions. An average of 7,761,850 reads per sample (50 bp) was generated (Supplementary Table S1). Raw sequencing reads were deposited at the Sequence Read Archive of NCBI (accession number SRA306211). Low quality ends (Q < 20) were trimmed and adapters were trimmed at the end when there was at least a 10 bp overlap and 90% match. Reads that were shorter than 35 bp after trimming were removed. Poly-A reads (more than 90% of the bases equal A), ambiguous reads (containing N), low quality reads (more than 50% of the bases Q < 25) and artefact reads (all but 3 bases in the read equal one base type) were removed. After processing, 99.8% of the initial reads remained. Processed reads were mapped to the reference *C. albicans* genome SC5314_A21 with Tophat v2.0.8b^65^. We removed reads from the alignment that are non-primary mappings or have a mapping quality ≤20. An average of 93.8% of the processed reads were mapped to the reference genome. The number of mapped reads that overlap with gene features (using the *C_albicansSC5314_version_A21_s02_m07_r10-features.gff* annotation file^66^) were determined with HTSeq 0.5.4p3^67^. Genes for which all samples have less than 1 count per million reads (absent genes) were not considered for differentially expressed gene analysis. For each of the 5,910 remaining genes, we performed differential expression analysis for each time point (i.e. 4 h and 24 h after miconazole treatment) with the EdgeR package of Bioconductor, by fitting a negative binomial generalized linear model^68^. The resulting *p* values were corrected for multiple testing with Benjamini-Hochberg to control the false discovery rate at 1%^69^. For identification of GO terms overrepresented in each of the differentially expressed gene lists, enrichment analysis was conducted with the *Candida* Genome Database GO Term Finder Tool^70^.

### Quantitative reverse transcription PCR

Primers for reverse transcription were designed using Primer-BLAST (www.ncbi.nlm.nih.gov/tools/primer-blast) (Supplementary Table S2). DNase treatment, reverse transcription and qRT-PCR analysis were carried out as described previously^71^. Two normalization genes, *ACT1* and *EFB1*, were used. Relative log2 induction ratios of treated samples compared with mock-treatment were calculated based on the ΔΔCt method^72^.

### BEC50 determination assay

To determine the minimal concentration of the compound that results in 50% remaining biofilm metabolic activity (BEC50) for the respective compounds, *C. albicans* SC5314 biofilms were grown and treated in a round bottomed microplate as described before^63^. Measurement of metabolic activity was performed using the metabolic activity dye CTB^63^.

### Biofilm checkerboard assay

In order to determine possible synergistic interactions between compounds, checkerboard analysis and FICI calculations were performed as described before using the metabolic activity dye CTB^63^ and FICI (fractional inhibitory concentration index) was calculated as FICI = [C_A_/BEC50_A_] + [C_B_/BEC50_B_], in which BEC50_A_ and BEC50_B_ are the BEC50 values of compound A and B alone, respectively, and C_A_ and C_B_ are the concentrations of the drugs A and B at iso-effective combinations, respectively^73^. Note that C_B_ values were derived by linear regression from the whole dose-response curves, whereas C_A_ values represent the actual concentration of compound A (i.e. simvastatin, pyrithione zinc, antimycin A, CCCP or sodium azide) in the checkerboard experiments, as indicated in Table 2. The interaction was defined as synergistic for a value of FICI ≤ 0.5, indifferent for 0.5 < FICI < 4 and antagonistic for FICI ≥ 4.0^74^.

### Planktonic assay

Synergistic action of compounds on planktonically growing *C. albicans* SC5314 cells was determined by FICI calculations as described above. MIC50 values (minimal concentration of the compound that causes a 50% reduction of planktonic cell growth), determined by OD measurement as described previously^75^ were used instead of BEC50 values. Sensitivity to miconazole (MIC50) was similarly assessed for planktonically growing ΔΔΔ*sod4sod5sod6* mutant and the corresponding isogenic wild-type strain, CA-IF100.

### Cfu determination and superoxide detection

Biofilms were grown and treated as described above. Alternatively, biofilms were grown as described above but the treatment was performed in anaerobic conditions by placing the biofilm plates in a jar in the Anoxomat^TM^ Mark II System (Advanced Instruments INC, Massachusetts, USA). After 24 h of treatment at 37 °C, biofilm cells were washed and dissolved in 100 μL PBS by vigorously pipetting. Of this cell suspension, 10 μL was diluted and plated out on YPD agar plates to determine the number of fungal cfu after 2 days of incubation at 30 °C. To determine the amount of superoxide formed, the remaining 90 μL was stained by adding 10 μL of a 200 μM dihydroethidium (Life Technologies Europe) stock solution. This suspension was transferred to black-walled microplates (Greiner) and incubated for 20 min at room temperature in the dark. Finally, fluorescence was measured with a fluorescence spectrometer (Synergy Mx multi-mode microplate reader, BioTek, USA) at λ_ex_ 510 nm and λe_em_ 595 nm and values were corrected for fluorescence measured in blank wells. Cfu determination was also performed for the ΔΔΔ*sod4sod5sod6* mutant and the corresponding isogenic wild-type strain, CA-IF100, after treatment with 30 μM miconazole. Calculation of the Pearson correlation coefficient and analysis of significance was performed in GraphPad Prism 6.

## Additional Information

**How to cite this article**: De Cremer, K. *et al.* Stimulation of superoxide production increases fungicidal action of miconazole against *Candida albicans* biofilms. *Sci. Rep.* **6**, 27463; doi: 10.1038/srep27463 (2016).

## Supplementary Material

Supplementary Information

Supplementary Dataset 1

Supplementary Dataset 2

## Figures and Tables

**Table 1:** Selection of inhibitors and inducers of the miconazole-affected pathways and combinatory action with miconazole against *C. albicans* biofilms determined by FICI calculation.

Process or protein	Effect in miconazole RNAseq data	Compound	BEC50	Activity in combination with miconazole^a^
		*Inhibitors*		
Sterol biosynthetic process	Induced	Simvastatin	1.4 mM	S
Secreted aspartic-type proteinases	Induced	Saquinavir mesylate	>500 μM	I
Lipases	Induced	Orlistat	>1 mM	I
Glyoxylate cycle	Induced	Itaconate	>2 mM	I
ETC	Repressed	Antimycin A	570 μM	S
		CCCP	73 μM	S
		Sodium azide	8.6 mM	S
Sphingolipid biosynthesis	Induced	Myriocin	>100 μM	I
Alcohol dehydrogenase	Induced	Disulfiram	>500 μM	I
Branched-chain amino acid (including leucine) biosynthesis	Induced	Chlorosulfuron	>1 mM	I
Fe-S bonds (as in Leu1 and many mitochondrial respiratory enzymes)	Induced	Pyrithione zinc	275 μM	S
Tetrahydrofolate interconversion	Induced	Trimethoprim	>500 μM	I
		*Inducers*		
Nicotinic acid transporter	Induced	Nicotinic acid	>1 mM	I
Arginine biosynthesis	Repressed	Arginine	>10 mM	I
Leucine biosynthesis	Induced	Leucine	>2 mM	I
Citric acid cycle	Repressed	Sodium dichloroacetate	>1 mM	I
N-acetylglucosamine transporter	Repressed	N-acetyl-D-glucosamine	>1 mM	I

^a^S = Synergy (FICI < 0.5), I = Indifference (0.5 < FICI < 4), FICI = [C_A_/BEC50_A_] + [C_B_/BEC50_B_], in which BEC50_A_ and BEC50_B_ are the BEC50 values (biofilm eradication concentration resulting in 50% remaining biofilm metabolic activity) of compound A and B alone, while C_A_ and C_B_ are the concentrations of the drugs A and B at iso-effective combinations, respectively. Note that C_B_ values were derived from the whole dose-response curves, whereas C_A_ values represent the actual concentration of compound A in the checkerboard experiments.

**Table 2:** Synergistic activity of simvastatin, pyrithione zinc, antimycin A, CCCP and sodium azide with miconazole against *C. albicans* biofilms.

Concentration compound (μM)	BEC50 miconazole (μM)^a^	Fold change^b^	FICI^c^
**Simvastatin**			
500	14	5.6	0.49
250	30	2.6	0.54
125	38	2.1	0.57
62.5	44	1.8	0.60
0	78	1.0	NA
**Pyrithione zinc**			
20	20	4.2	0.31
10	19	4.4	0.26
5	24	3.5	0.30
2.5	52	1.6	0.63
0	84	1.0	NA
**Antimycin A**			
25	6.4	12.7	0.12
12.5	7.5	10.8	0.11
6.25	9.9	8.2	0.13
3.125	7.4	10.9	0.10
0	81	1.0	NA
**CCCP**			
62.5	7.7	13.6	0.93
31.25	18	5.8	0.60
15.625	27	3.9	0.47
7.8125	62	1.7	0.70
0	105	1.0	NA
**Sodium azide**			
5,000	8.8	6.8	0.73
2,500	16	3.8	0.56
1,250	22	2.7	0.51
625	25	2.4	0.49
0	64	1.0	NA

The BEC50 values are the mean ± SEM of at least five (n = 5) independent biological replicates (each consisting of three repeats). FICI values indicating a synergistic interaction are printed in bold.

^a^BEC50 = Biofilm eradication concentration resulting in 50% remaining biofilm metabolic activity, calculated using nonlinear regression. The BEC50 value of simvastatin, pyrithione zinc, antimycin A, CCCP and sodium azide is 1.6 mM, 275 μM, 570 μM, 73 μM and 8.6 mM, respectively.

^b^Fold change = fold increase of biofilm activity of miconazole due to the combination, calculated as (BEC50 of miconazole alone)/(BEC50 of miconazole in combination).

^c^FICI = Fractional inhibitory concentration index, NA = Not Applicable.
