# Supplementary material for: Stimulation of superoxide production increases fungicidal action of miconazole against Candida albicans biofilms
Source: Sci Rep. 2016 Jun 7;6:27463. doi: 10.1038/srep27463 (PMC4895440; doi:10.1038/srep27463)
Supplement: Supplementary Information [file srep27463-s1.pdf]

Stimulation of superoxide production increases fungicidal action of miconazole against *Candida albicans* biofilms

Kaat De Cremer[1,2], Katrijn De Brucker[1], Ines Staes[1], Annelies Peeters[1], Freija Van den Driessche[3], Tom Coenye[3], Bruno P.A. Cammue[1,2*] and Karin Thevissen[1]

Supplementary Information

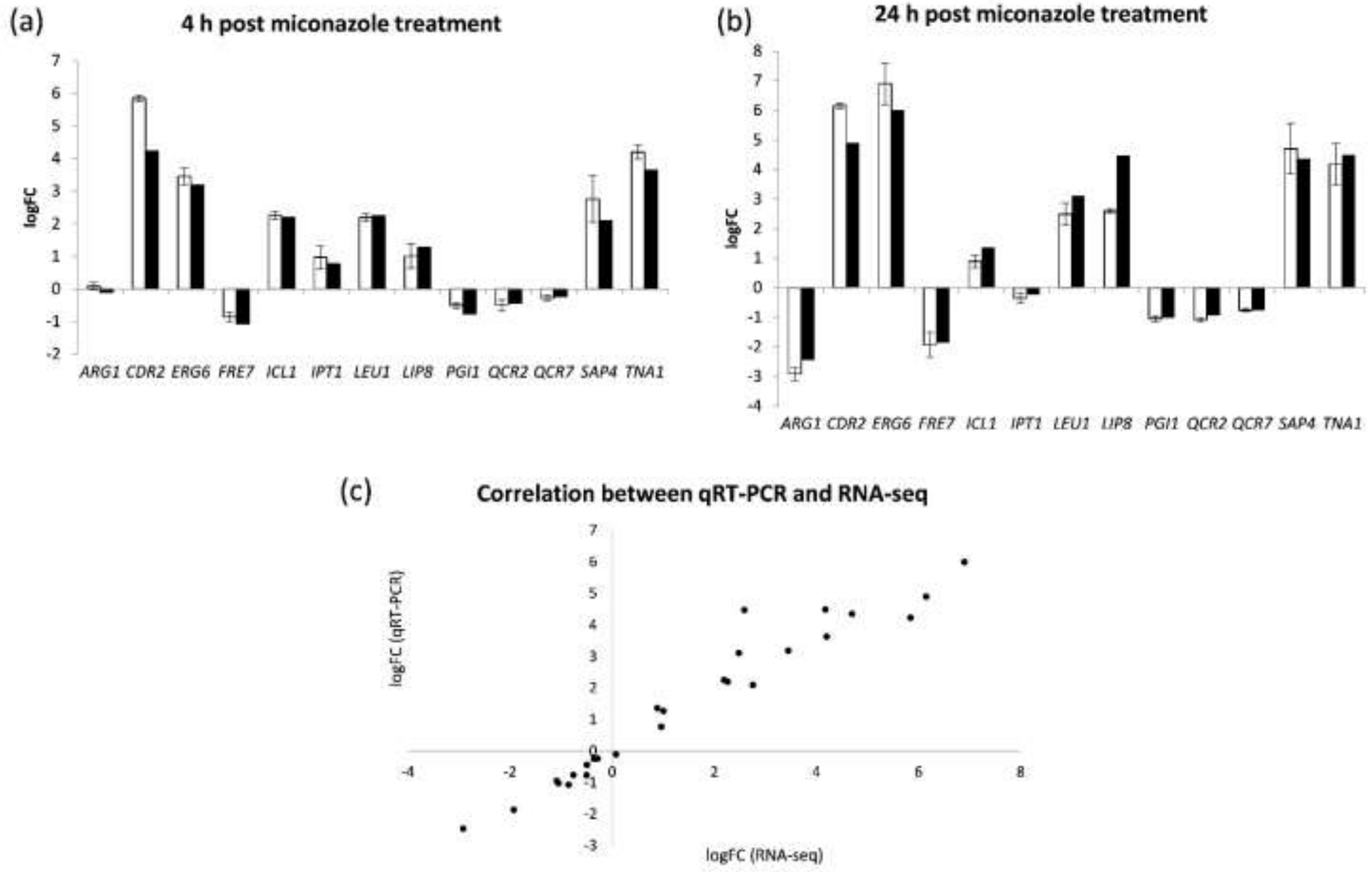

Figure S1. Validation of RNA-seq results using qRT-PCR. The log fold change (logFC) for a selected set of genes in *C. albicans* biofilms 4 h (a) and 24 h (b) after treatment with miconazole calculated with qRT-PCR and RNA-seq. White bar: qRT-PCR results, data are represented as log2 ratios (treatment/control) ± SEM from three independent biological samples; Black bar: RNA-seq logFC ratio as calculated by EdgeR. (c) Correlation between both methodologies.

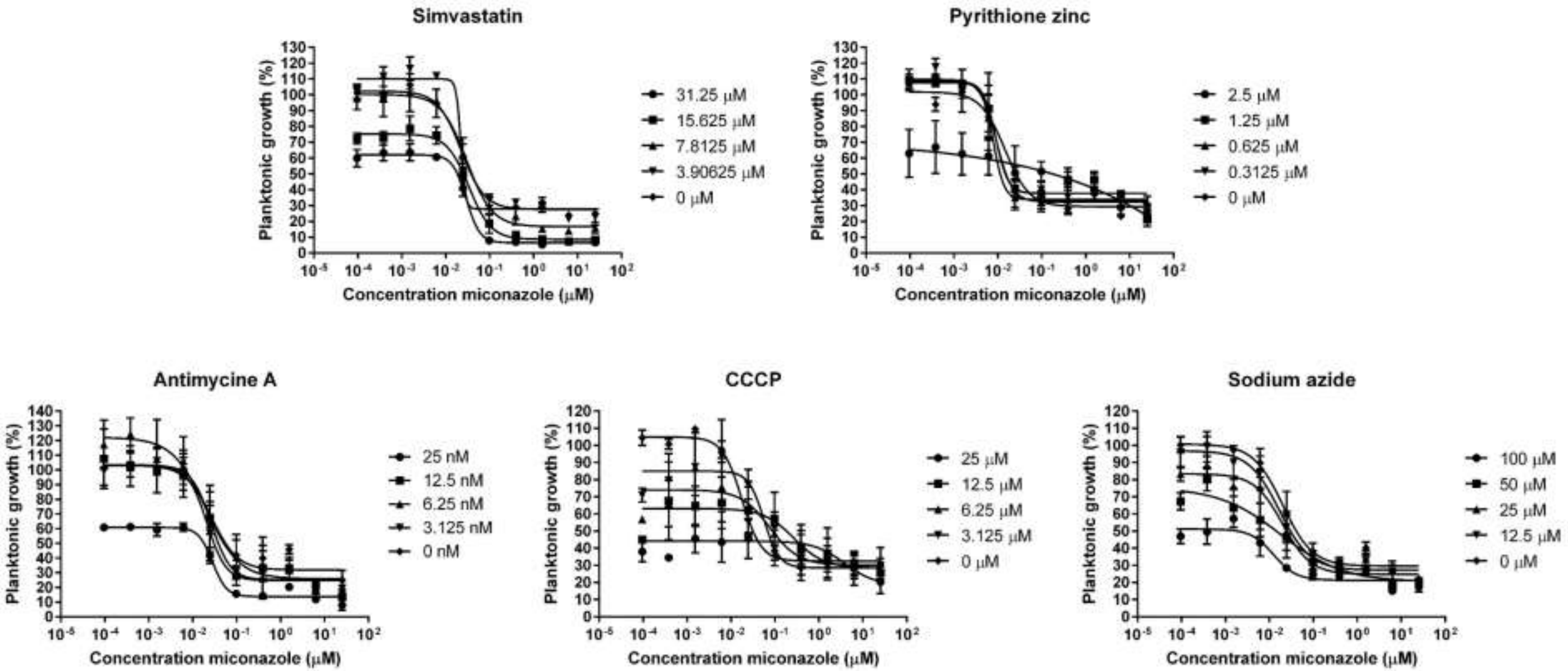

Figure S2. Growth of *C. albicans* planktonic cultures in the presence of a combination of miconazole and simvastatin, pyrithione zinc, antimycin A, CCCP and sodium azide. The control curve of miconazole alone is represented by diamonds, whereas combinations of miconazole with decreasing concentrations of other compound are indicated by circles, crosses, squares, triangles and reverted triangles. Data are mean ± SEM of three biologically independent experiments consisting of triplicate measurements (n=3), determined by OD measurement.

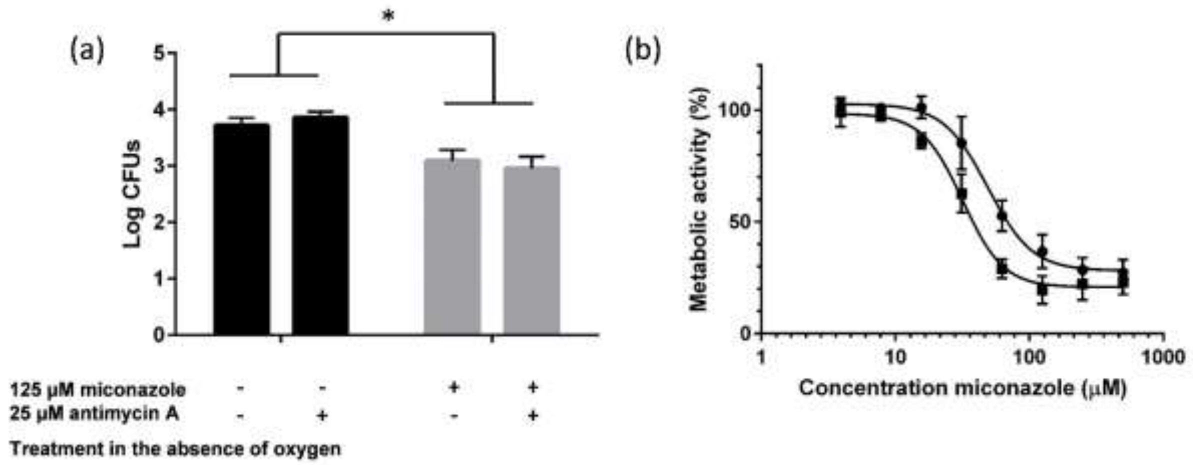

Figure S3. Number of cfus (a) per *C. albicans* biofilm treated with miconazole and/or antimycin A in the absence of oxygen and metabolic activity (b) of *C. albicans* biofilms treated with miconazole in aerobe and anaerobe conditions. (a) Counting of cfus was performed in a plating assay. Black bars represent untreated biofilms or biofilms treated with 25 µM antimycin A alone while grey bars represent biofilms additionally treated with 125 µM miconazole. Data presented are the mean and SEM of five ($n = 5$) independent experiments (each consisting of three replicates). Two-way ANOVA statistical analysis with Tukey's correction for multiple comparisons was performed to assign significant differences between treatments (* = $p < 0.05$). (b) Treatment in aerobe and anaerobe conditions is represented by circles and squares, respectively. Data are mean ± SEM of four biologically independent experiments (each consisting of three repeats), as determined with the metabolic activity dye CTB.

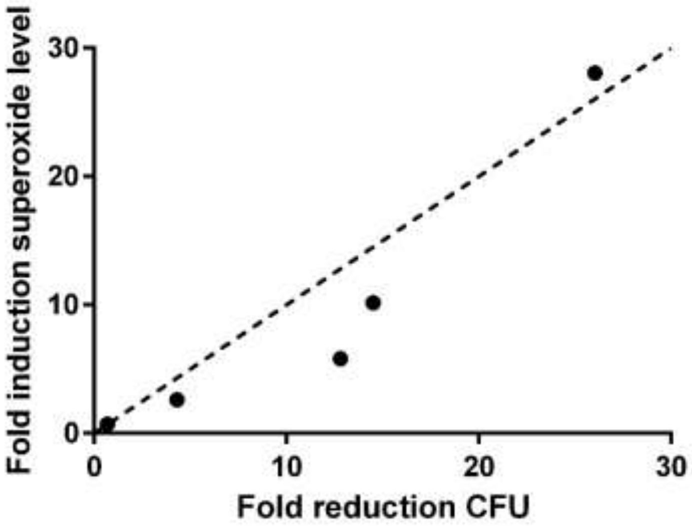

Figure S4. Correlation between the level of superoxide radical induction and reduction in cfus for treated biofilms. The samples considered for this analysis are *C. albicans* biofilms treated with miconazole alone and treatment with a combination of miconazole and inhibitor (simvastatin, pyrithione zinc, antimycin A, CCCP and sodium azide). To simplify visual interpretation, the first bisector is presented as a dashed line.

Table S1. Number of reads per sample that were sequenced and (uniquely) mapped to the reference genome.

	sequenced reads (#)	mapped reads (#)	uniquely aligned reads (#)
**Control 4 h A**	8,839,867	8,239,088	7,142,368
**Control 4 h B**	8,827,978	8,194,960	6,913,430
**Control 4 h C**	8,687,442	8,089,149	6,816,172
**Miconazole 4 h A**	8,511,295	7,939,157	6,786,650
**Miconazole 4 h B**	8,937,806	8,305,708	6,973,904
**Miconazole 4 h C**	8,679,355	8,095,024	6,760,119
**Control 24 h A**	9,380,590	8,755,400	7,389,317
**Control 24 h B**	7,005,190	6,529,533	5,214,235
**Control 24 h C**	7,138,416	6,654,512	5,530,500
**Miconazole 24 h A**	8,072,929	7,528,765	6,118,106
**Miconazole 24 h B**	8,163,592	7,651,999	6,102,525
**Miconazole 24 h C**	7,655,584	7,158,906	5,770,898
**Total**	99,900,044	93,142,201	77,518,224
**Average**	8,325,004	7,761,850	6,459,852

Table S2. Primers used in this study.

Gene ID	Gene name	Sequence 5' → 3' (forward/reverse)
orf19.7469	*ARG1*	GTTTTGCCGTTTCTCACGGTT/GTTTGTGCCACTGGGATGTTT
orf19.5958	*CDR2*	CCTGGAAGCACAGTTGTCCA/TCCCCCTTTTGCATAGCACC
orf19.1631	*ERG6*	GTGGTGTAGGTGGTCCTGGTA/AAAACTGGAGCATGAACGGTA
orf19.6139	*FRE7*	GCCAATTAGGGTCGGTGTGG/CCAATCTCCGTTTGCACCCT
orf19.6844	*ICL1*	AGATTGGTTGCCATCAGAGCC/TCCGGCTTCTGGGTTAGTGG
orf19.4769	*IPT1*	CGTGAACCAATACGATTATGGCAA/AAGGTGCAGCATTGGGGAATA
orf19.7498	*LEU1*	GCTCCAAAGGGACAAGAATGGG/GTTGCTGGGTCTGGGACACT
orf19.1345	*LIP8*	AATCCAGGGAAACCCCAAAGTC/TGATAAGAAGCAACTTTGGAAGGG
orf19.3888	*PGI1*	ACCAGCCACTAACGCTCAAC/TGGGCAAAGAAGTTGGAAGCC
orf19.2644	*QCR2*	TCCAACCAACACTTTCACTGGTC/AAGTAGCACCTGGAATTTGTGCT
orf19.5629	*QCR7*	TGTCGTTAAGGCAGCTAATTTCAT/ATAGCAGTTTGCATAATTGGGGTT
orf19.5716	*SAP4*	CTACAGGGTTTGTTACCTTAGACTT/TCCTCTTTTGCCCACATCATTTCTA
orf19.4335	*TNA1*	TTGGGTGCAACTCATAGCCC/CCAATTTGCCAAGCAGTACCC
orf19.5007	*ACT1*	TCGATAACGGTTCTGGTATGTGT/TTTTGGATTGGGCTTCATCACC
orf19.3838	*EFB1*	GAACGAATTCTTGGCTGACAAATCA/AAGCGGCTGGGGCTTTAC

Data S1. Gene lists of all/DE genes that were detected with RNA-seq at 4 h or 24 h after miconazole treatment. DE gene analysis between miconazole-treated and untreated samples at both time points was performed using EdgeR, applying a limited false discovery rate (FDR adjusted p-value < 0.01).

Data S2. All significantly overrepresented GO categories within the significantly up- or down-regulated gene lists at 4 h and 24 h post miconazole treatment, respectively. Significant overrepresented GO categories were determined using the GO Term Finder of the Candida genome Database (FDR adjusted p-value < 0.05).
